# Prevalence and detection of psychosocial problems in cancer genetic counseling

**DOI:** 10.1007/s10689-015-9809-9

**Published:** 2015-05-13

**Authors:** W. Eijzenga, E. M. A. Bleiker, D. E. E. Hahn, L. E. Van der Kolk, G. N. Sidharta, N. K. Aaronson

**Affiliations:** Division of Psychosocial Research and Epidemiology, The Netherlands Cancer Institute, Plesmanlaan 121, 1066 CX Amsterdam, The Netherlands; Family Cancer Clinic, The Netherlands Cancer Institute, Amsterdam, The Netherlands; Department of Psychosocial Counseling, The Netherlands Cancer Institute, Amsterdam, The Netherlands

**Keywords:** Hereditary cancer, Psychosocial problems, Genetic counseling, Risk factors, Distress, Oncology

## Abstract

Only a minority of individuals who undergo cancer genetic counseling experience heightened levels of psychological distress, but many more experience a range of cancer genetic-specific psychosocial problems. The aim of this study was to estimate the prevalence of such psychosocial problems, and to identify possible demographic and clinical variables associated significantly with them. Consenting individuals scheduled to undergo cancer genetic counseling completed the Psychosocial Aspects of Hereditary Cancer (PAHC) questionnaire, the Hospital Anxiety and Depression Scale (HADS) and the Distress Thermometer (DT) prior to or immediately following their counseling session. More than half of the 137 participants reported problems on three or more domains of the PAHC, most often in the domains ‘living with cancer’ (84 %), ‘family issues’ (46 %), ‘hereditary predisposition’ (45 %), and ‘child-related issues’ (42 %). Correlations between the PAHC, the HADS and the DT were low. Previous contact with a psychosocial worker, and having a personal history of cancer were associated significantly with HADS scores, but explained little variance (9 %). No background variables were associated significantly with the DT. Previous contact with a psychosocial worker, and having children were significantly associated with several PAHC domains, again explaining only a small percentage of the variance (2–14 %). The majority of counselees experience specific cancer genetic counseling-related psychosocial problems. Only a few background variables are associated significantly with distress or psychosocial problems. Thus we recommend using the PAHC or a similar problem-oriented questionnaire routinely in cancer genetic counseling to identify individuals with such problems.

## Introduction

One of the main messages of studies on the psychosocial impact of genetic counseling for cancer is that, after the process of genetic counseling and risk assessment has been completed, distress levels for the majority of counselees return to or are even lower than baseline levels [1–3]. However, approximately one-quarter of counselees experience heightened levels of distress during and/or after the genetic counseling process [4].

The psychosocial impact of genetic counseling is most frequently measured with the Hospital Anxiety and Depression scale (HADS), the State Trait Anxiety Inventory, the Impact of Event Scale, or the Center for Epidemiological Studies Depression Scale [5–7]. However, these questionnaires may be too generic to capture the entire spectrum of psychosocial issues relevant to the cancer genetic setting [8]. They do not capture other important issues and concerns, such as existential problems, family related problems, issues surrounding genetic risk, the burden of living with cancer, and possible practical problems related to genetic counseling (e.g., insurance issues) [8–10].

Several methods are available to assist genetic counselors in detecting counselees with serious psychosocial problems. It has been proposed to use sociodemographic and clinical risk factors to identify (potentially) distressed individuals [11, 12]. Vadaparampil et al. [7] recommend inquiring routinely about previous contacts with psychosocial caregivers as a means of identifying counselees potentially in need of such services.

Increasingly, the Distress Thermometer (DT) with an accompanying problem checklist is being recommended as a first line screening method for distress in daily clinical oncology practice [13]. The DT, together with a revised checklist designed specifically for women at high risk of developing breast cancer has proven to be useful in screening for distress at the time women undergo mammography [14].

Recently, we developed the Psychosocial Aspects of Hereditary Cancer (PAHC) questionnaire as a tool for identifying psychosocial issues and concerns experienced during cancer genetic counseling [15]. The PAHC questionnaire consists of 26 items, organized into six domains. We have established a threshold per domain of the PAHC questionnaire for identifying counselees who may need further psychosocial care [15].

Knowledge of the specific psychosocial problems and distress levels experienced by counselees, as well as factors that may be associated with such problems can provide genetic counselors with useful information that they can use during the genetic counseling session. In this paper, we report on a study of the prevalence of cancer genetic counseling-specific psychosocial problems and their association with more generalized distress as assessed by the HADS and the DT. We also investigated whether sociodemographic and clinical variables are associated significantly with psychosocial problems and psychological distress experienced during cancer genetic counseling.

## Materials and methods

The data reported here were collected as a part of a larger study that evaluated the screening properties of the PAHC questionnaire and the DT in the cancer genetic counseling setting [15]. The institutional review board of the hospital approved this study, and informed consent was obtained from all individual participants included in the study.

### Participants

Individuals were eligible to participate when they were scheduled for a visit at the family cancer clinic of The Netherlands Cancer Institute to undergo genetic counseling for any type of hereditary cancer syndrome in the period January–December, 2010, were over 18 years of age, and had a sufficient command of the Dutch language.

### Procedure

Eligible counselees received a letter of invitation from the head of the family cancer clinic and, if interested, were requested to return a signed consent form by mail. A reminder letter was sent 1 week before the genetic counseling session. Participants completed a questionnaire on a touchscreen computer at the clinic with demographic questions, the PAHC questionnaire, the DT and the HADS. The preference was to have the questionnaire completed prior to the counseling, but this was not always feasible due to planning issues. Thus counselees completed the questionnaire immediately prior to their scheduled genetic counseling session or immediately thereafter.

### Sociodemographic and clinical data

The counselees’ age, sex, marital status, education level, number of children, the number of affected first degree relatives, and use of psychosocial services in the past for any problem (i.e., not necessarily in the cancer genetic counseling setting) were obtained via self-report. Data on whether (s)he was diagnosed with cancer in the past and, if so, at what age, and whether there was a known gene mutation in the family were extracted from the medical records.

### The PAHC questionnaire

The PAHC questionnaire consists of 26 questions addressing psychosocial problems and concerns that are specifically relevant to counselees within the cancer genetic counseling and testing setting. The content of the PAHC questionnaire is organized into the following six domains: (1) hereditary predisposition; (2) practical issues; (3) family and social issues; (4) general emotions; (5) living with cancer; and, for those who have children (6) children-related issues. The number of items per domain varies between 2 and 6. All 26 items are scored on a 4-point, Likert-type scale ranging from 1 (“not at all”) to 4 (“very much”). Based on a detailed analysis of the screening properties of the PAHC questionnaire, a threshold was established for clinical relevance [15]. Specifically, if one or more items within a domain is rated with a 3 or a 4 (i.e., indicating a moderate to severe problem), that domain is considered as a positive case. Additionally, per problem domain, the respondent is asked to indicate whether (s)he would like to receive professional psychosocial support. The PAHC questionnaire is supplemented by the DT, a visual analogue scale ranging from 0 to 10 (no distress to severe distress) [13]. The timeframe of the PAHC questionnaire and the DT is the previous week.

### The HADS

The HADS was used to assess general psychological distress. It includes 14 questions and yields a total score and subscale scores for anxiety and depression. In the current analysis, we used only the total score, with a possible range of 0–42. Higher scores represent higher levels of distress. The HADS has been validated for use in the Netherlands [16].

### Statistical analysis

We used analysis of variance and Chi square analyses to compare study participants and non-participants on sociodemographic and clinical characteristics. Chi square analysis and Student’s *t* tests were used to examine potential differences in responses to the PAHC questionnaire, the HADS and the DT as a function of timing of questionnaire completion (i.e., prior to or immediately following the counseling session). The association between the PAHC questionnaire domains, the HADS and the DT was assessed by calculating Pearson’s correlation coefficients and partial correlations that controlled for inter-correlations between the domains of the PAHC questionnaire.

Chi square and Student’s *t* tests were employed to investigate which sociodemographic and clinical variables, if any, were associated significantly with the PAHC questionnaire domains, the HADS, and the DT. Any variable with a *p* value below 0.10 was entered subsequently into a logistic (for the PAHC domain scores) or a linear regression model (for the HADS, and the DT). Only those participants with children completed the domain addressing children-related issues. Thus the analyses relating to this domain were performed on the subgroup of participants with children (n = 100).

## Results

### Participants

In total, 263 eligible counselees were invited to participate in the study, of whom 139 (53 %) agreed to do so. Reasons for non-participation included logistical or scheduling problems (n = 23), perceived emotional burden (n = 20), lack of interest (n = 13), and not wanting the counseling session to be audiotaped (n = 3) (audiotaping was employed for another part of the study). Thirty-nine counselees provided other reasons, and 26 did not provide a reason. Two additional cases were excluded from the analysis because their clinical data were not available. This resulted in a total of 137 cases for the analysis. No statistically significant differences were observed between study participants and non-participants on any of the available sociodemographic and clinical variables.

The sociodemographic characteristics of the sample are reported in Table 1. The mean age of the sample was 47.1 years (range 18–78), and the large majority was female and being counseled for hereditary breast and ovarian cancer syndrome (82 %). Most respondents were married or in a steady relationship, had children, and reported that they were not aware of any DNA-mutation in the family. Approximately half of the sample was relatively highly educated, had had contact with a psychologist or social worker at some time in the past, and had previously been diagnosed with cancer. There were no statistical significant differences on any of these background variables between those who completed the questionnaires before (n = 91) or after (n = 46) the genetic counseling session.

**Table 1:** Sociodemographic and clinical characteristics of the study sample (n = 137)

	Participants (n = 137)
Age (years) [SD]	47.1 [11.3]

	N (%)
*Sex*	
Male	25 (18)
Female	112 (82)
*Marital status*	
Married/steady relationship	123 (90)
Single/divorced/widow/widower	14 (10)
*Education level* ^a^	
Low	31 (23)
Middle	43 (32)
High	62 (46)
*Children*	
Yes	100 (73)
No	37 (27)
*Previous contact with psychosocial worker*	
Yes	69 (50)
No	68 (50)
*First in family being referred to cancer genetic counseling*	
Yes	87 (64)
No	50 (36)
*Mutation in family before counseling*	
Yes	33 (24)
No	104 (76)
*Personal history of cancer*	
Yes	71 (52)
No	66 (48)

^a^n = 136, one participant had an unknown educational level

### Prevalence of psychosocial problems and their relation to distress

Approximately 10 % of the participants did not report any problems included in the PAHC questionnaire that were of a sufficient magnitude (i.e., a score of 3 or 4 on an item within any given domain) to be considered relevant for further discussion. Fifty-four percent of the participants met the threshold for clinical relevance on three or more domains of the PAHC questionnaire (Table 2). The domain with the highest prevalence was ‘living with cancer’ (84 %), followed by ‘hereditary predisposition’ (46 %), ‘family and social issues’ (45 %), and ‘child-related issues’ (42 %). The domains ‘general emotions’ (29 %), and ‘practical issues’ (19 %) had the lowest prevalence in our sample (Table 3).

**Table 2:** Frequency and percentages of PAHC questionnaire domains with scores above the threshold

	Frequency (n = 137)	Percentage	Cumulative percentage
None	14	10.2	10.2
1 domain	30	21.9	32.1
2 domains	19	13.9	46.0
3 domains	27	19.7	65.7
4 domains	27	19.7	85.4
5 domains	15	10.9	96.4
6 domains	5	3.6	100

**Table 3:** Percentage of counselees with PAHC questionnaire scores above the threshold for clinical relevance per domain and correlations with the HADS and DT^a^

Domain	Above the threshold (%) (n = 137)	HADS^b^		DT^c^	
		Pearson’s correlation	Partial correlation^d^	Pearson’s correlation	Partial correlation^d^
Hereditary predisposition	46	0.33**	0.16	0.31**	0.24**
Practical issues	19	0.23**	0.09	0.26**	0.17*
Family and social issues	45	0.33**	0.19*	0.16	0.03
General emotions	29	0.54**	0.49***	0.29**	0.25**
Living with cancer	84	0.29**	0.14	0.14	0.02
Child-related issues	42	0.24**	−0.05	0.09	−0.10

*HADS* Hospital Anxiety and Depression Scale, *DT* Distress Thermometer

* *p* < 0.05; ** *p* < 0.01; *** *p* < 0.001

^a^Pearson’s correlation between HADS and DT = 0.58***

^b^Distress as measured with the HADS, adjusted R square of the model = 0.37

^c^Distress as measured with the DT, adjusted R square of the model = 0.15

^d^Association between variables controlling for inter-correlation between the domains

All of the PAHC questionnaire domains were correlated significantly with psychological distress as measured by the HADS, when based on a Pearson correlation coefficient. However, when correcting for inter-domain correlations, only the domains ‘family and social issues’ and ‘general emotions’ remained statistically significantly associated with the HADS. All of the partial correlations were low, with the exception of the domain ‘general emotion,’ which has a strong conceptual overlap with distress as assessed by the HADS (Table 3).

The domains ‘hereditary predisposition’, ‘practical issues’, and ‘general emotions’ had statistical significant Pearson’s correlations with distress as measured by the DT. These domains remained statistically significant when correcting for inter-domain correlations. However, the magnitude of the (partial) correlations was relatively low (Table 3).

### Sociodemographic and clinical variables associated with general distress

Education level, having had previous contact with a psychosocial worker, and having a personal history of cancer were associated significantly with general distress as measured by the HADS (see Table 4). When entered in a linear regression model, only having had previous contact with a psychosocial worker (*p* = 0.001), and having a personal history of cancer (*p* = 0.03) remained statistically significant. However, only 10 % of the variance in distress scores was explained by these three variables.

**Table 4:** Sociodemographic and clinical variables associated with general distress, assessed with the HADS and the DT

	B (SE)	exp b	95 % CI for B	
			Lower	Upper
*HADS* ^a^				
Education level	−0.10 (0.68)	−0.01	−1.45	1.26
Previous contact with psychosocial worker	3.61 (1.08)**	0.28	1.48	5.74
Personal history of cancer	2.45 (1.09)*	0.19	0.31	4.60
*DT* ^b^				
Marital status	1.11 (0.77)	0.12	−0.40	2.64
Previous contact with psychosocial worker	0.79 (0.47)	0.14	−0.13	1.71
Known mutation in family	−0.52 (0.58)	−0.08	−1.67	0.63
Personal history of cancer	0.73 (0.50)	0.13	−0.25	1.71

*HADS* Hospital Anxiety and Depression Scale, *DT* Distress Thermometer

* *p* < 0.05; ** *p* < 0.01

^a^Adjusted R square of the model = 0.10

^b^Adjusted R square of the model = 0.08

Marital status, having had previous contact with a psychosocial worker, having a known mutation in the family, and having a personal history of cancer were statistically significantly associated with the DT. However, none of these variables remained statistically significant when entered in a linear regression model. The variance in distress scores explained by these four variables was 8 %.

### Sociodemographic and clinical variables associated with PAHC questionnaire domains

At the univariate level, the following statistically significant associations were observed between background variables and the PAHC questionnaire domains: having children with the domain ‘hereditary predisposition’; age and having had previous contact with a psychosocial worker with the domain ‘practical issues’; having children, being the first in the family to undergo genetic counseling, and sex with the domain ‘family and social issues’; having had previous contact with a psychosocial worker and having a personal history of cancer with the domain ‘general emotions’; and sex, the total number of children, and a known DNA-mutation in the family with the domain ‘living with cancer’.

At the multivariate level, having children was the only variable associated significantly with the domains ‘hereditary predisposition’ (*p* = 0.02) and ‘family and social issues’ (*p* = 0.007). Previous contact with a psychosocial worker (at any time in the past, for any problem) was associated significantly with the domain ‘practical issues’ (*p* = 0.04). No sociodemographic or clinical variables exhibited statistically significant associations with the domains ‘general emotions’, ‘living with cancer’ or ‘child-related issues’. The variance in the PAHC domain scores explained by these regression models ranged from 2 to 14 % (Table 5).

**Table 5:** Sociodemographic and clinical variables associated with PAHC questionnaire domains

	B(SE)	exp b	95 % CI for exp b		Nagelkerke R square
			Lower	Upper	
*Hereditary predisposition*					0.05
Having children	0.94 (0.41)*	2.56	1.14	5.74	
Constant	−0.86 (0.36)*	0.42			
*Practical issues*					0.10
Age	−0.37 (0.02)	0.96	0.93	1.00	
Previous contact with psychosocial worker	−0.97 (0.47)*	0.38	0.15	0.96	
*Family and social issues*					0.14
Having children	1.27 (0.47)**	3.56	1.41	8.94	
First in family to undergo genetic counseling	0.72 (0.39)	2.06	0.96	4.39	
Sex	−0.33 (0.54)	0.72	0.25	2.06	
*General emotions*					0.06
Previous contact with psychosocial worker	−0.57 (0.39)	0.57	0.27	1.21	
Personal history of cancer	−0.75 (0.39)	0.47	0.22	1.02	
*Living with cancer*					0.02
Sex	−0.35 (0.87)	0.71	0.13	3.91	
Total number of children	0.29 (0.38)	1.33	0.64	2.79	
Known mutation in family	0.53 (0.68)	1.70	0.45	6.42	

The domain of ‘child-related issues’ did not yield any statistical significant factors

*PAHC* Psychosocial Aspects of Hereditary Cancer questionnaire

* *p* < 0.05; ** *p* < 0.01

## Discussion

In this paper we have reported on the prevalence of specific psychosocial problems experienced by counselees at the time that they attended a family cancer clinic for their first cancer genetic counseling session. Many counselees reported moderate to severe problems in the various domains assessed by the PAHC questionnaire, such as ‘living with cancer’, ‘hereditary predisposition’, ‘family and social issues’, and ‘child-related problems’. These results are in line with those reported by Bennett et al. [17] who, using a different questionnaire, found that up to two-thirds of counselees experienced concerns related to the impact of genetic counseling and testing on family members. In our study, 54 % of counselees reported problems on at least three different PAHC questionnaire domains of sufficient severity to merit discussion with the genetic counselor. It is important that such problems are detected and discussed during genetic counseling [18, 19], as that can lead to an improved relationship between counselor and counselee, and may ultimately may result in the resolution of those problems and of associated distress [20].

Some investigators have proposed using sociodemographic and clinical risk factors or risk profiles to identify individuals who are likely to be(come) distressed [11, 12]. Although we identified some variables that are associated significantly with both generalized distress and specific cancer genetic-specific problems, the percentage of variance explained by these variables was consistently low. This suggests that sociodemographic and clinical variables may not be particularly useful in identifying particularly vulnerable counselees. Rather, such background variables can be used as probes once a counselee reports being distressed and/or having specific psychosocial problems related to the genetic counseling process. For example, if a counselee reports family and social issues at the time of counseling, the counselor can inquire further about the potential role of having children and of being the first in the family being referred to genetic counseling.

We would stress the potential importance of asking counselees about their specific psychosocial problems at the time of cancer genetic counseling, prior to undergoing DNA testing and receiving the DNA test results. Studies of the routine use of patient-reported outcome measures in daily clinical oncology practice have demonstrated their value in enhancing communication between patients and their health care providers [21–25]. We have conducted a randomized controlled trial, using the PAHC questionnaire, which showed the promising potential of the questionnaire as a valuable first-line screening instrument in the cancer clinical genetics setting [26–28].

There are several limitations of the current study that should be noted. First, only 53 % of those invited to participate in the study actually did so. Although we did not observe any statistically significant differences between participants and non-participants on sociodemographic or clinical background variables, we cannot say with certainty that our sample was entirely representative of the larger population of interest. However, while a small minority of the non-participants (20 of 124 = 16 %) indicated that they thought the study would be too emotionally burdensome for them (suggesting underling psychosocial problems and/or distress), the majority of non-participants either reported more neutral reasons (e.g., logistical problems, lack of interest, not wanting to be audiotaped, etc.) or did not provide a reason. Second, the large majority of study participants was female and was being counseled for hereditary breast and ovarian cancer, reflecting the population of counselees attending the family cancer clinical at the Netherlands Cancer Institute. However, our results cannot necessarily be generalized to those with other hereditary syndromes. As we did not have sufficient statistical power to do so, future studies are needed to determine if the prevalence of psychosocial problems varies significantly as a function of hereditary cancer syndrome and of sex. Third, the PAHC questionnaire was administered either prior to or immediately following the genetic counseling session. This could potentially affect the observed prevalence of psychosocial problems and the associations observed between the PAHC questionnaire and the HADS and DT, and between the PAHC questionnaire and various sociodemographic and clinical variables. However, our analyses indicated that the prevalence of psychosocial problems did not vary significantly as a function of the timing of the questionnaire administration. Fourth, the domains of the PAHC questionnaire were correlated. While this could potentially complicate the interpretation of observed correlations between the PAHC and other measures and variables, the use of partial correlations corrected for this.

The study also had several important strengths. First, as indicated above, the study sample was representative of the population undergoing genetic counseling in our clinic. Second, we included a range of sociodemographic and clinical variables that have been frequently used to try to identify those at risk for psychosocial problems and psychological distress. Thus we were able to compare directly the relative value of risk profiles based on background variables with a psychosocial screening questionnaire in identifying those with clinically relevant psychosocial problems.

In conclusion, although only a minority of individuals who undergo cancer genetic counseling suffer from high levels of psychological distress, the large majority reports a range of psychosocial problems related specifically to cancer genetic counseling. The PAHC questionnaire is a useful tool for identifying relevant psychosocial problems that merit further attention in clinical practice. Use of such a tool can contribute significantly to enhancing the quality of communication between genetic counselors and their clients, to providing client-centered care, and to addressing relevant psychosocial problems in a timely manner.
